# The omphalomesenteric duct fibrous band as a rare cause of bowel obstruction: A case report and literature review

**DOI:** 10.1016/j.ijscr.2024.109354

**Published:** 2024-02-03

**Authors:** Hamed Tahmasbi, Alireza Haghbin Toutounchi, Arman Hasanzade, Shaghayegh Sadeghmousavi, Mohammad Aghaei

**Affiliations:** Department of General Surgery, Imam Hossein Medical and Educational Center, Shahid Beheshti University of Medical Sciences, Tehran, Iran

**Keywords:** Omphalomesenteric duct, Intestinal obstruction, Bowel obstruction, Meckel's diverticulum, Fibrous band

## Abstract

**Introduction and importance:**

The omphalomesenteric duct (OMD) is an embryonic structure that normally undergoes obliteration during embryonic development, typically not persisting after birth. Failure of complete or partial obliteration can result in a type of malformation known as OMD remnant.

**Case presentation:**

We report a case of a 24-year-old male patient diagnosed with bowel obstruction. Abdominal computed tomography (CT) scan revealed the presence of an adhesion band. During surgery, a fibrous band connecting from the umbilicus to the mesentery of terminal ileum was found and resected. Pathological investigation confirmed the presence of an OMD remnant fibrous band.

**Clinical discussion:**

OMD remnant can manifest in different forms such as Meckel's diverticulum, umbilical polyp, OMD cyst, OMD fistula, and fibrous band, occurring in approximately 2 % of infants and often presenting symptoms in early childhood. These conditions rarely cause complications in adults. Complications may include obstruction, gastrointestinal bleeding, bowel perforation, and omphalitis which can present with symptoms such as abdominal pain, vomiting, melena, lack of defecation, umbilical discharge, and dermal manifestations. Diagnostic approaches vary depending on the type of OMD remnant and associated complications, but ultrasonography and CT scan can be useful. While asymptomatic OMD remnants generally do not require further intervention, surgical treatment is the main option for complicated and symptomatic cases.

**Conclusion:**

OMD remnant is a rare condition in adults that can lead to complications. Given that obstruction is a common complication of OMD remnant, OMD remnant should be considered in the differential diagnosis of patients presenting with bowel obstruction.

## Highlights

• Symptomatic omphalomesenteric duct remnants are exceedingly rare in adults.
• Diverse symptoms of omphalomesenteric duct remnants make the diagnosis challenging.
• Omphalomesenteric duct remnant causes bowel obstruction with different mechanism.

## 1 Introduction

The Omphalomesenteric Duct (OMD), also known as the Vitelline duct, is a structure that connects the embryonic midgut to the primary yolk sac. This duct is obliterated approximately between the 7th and 10th weeks of gestation. However, in cases where the duct fails to obliterate, it can result in a congenital anomaly known as OMD remnant d [1]. Various types of OMD remnants exist, but they are considered rare, affecting approximately 2 % of the population [2,3]. These types include Meckel diverticulum, OMD cyst, fistula, umbilical polyp, and fibrotic band [4]. OMD remnants typically do not cause any symptoms and often go unnoticed unless complications arise, such as hemorrhage, obstruction, intussusception, inflammation, or perforation. These complications primarily occur in the pediatric population, particularly within the first year of life. As a result, symptomatic OMD remnants in adults are uncommon [1,2]. In this paper, we present a case of an OMD remnant with a fibrous-band caused obstruction in a 24-year-old male adult. This article has been reported in line with the SCARE 2023 criteria [5].

## 2 Presentation of case

### 2.1 History

A 24-year-old immigrant male adult presented to the emergency room with complaints of abdominal pain. The pain had started two days before and had progressively worsened. Initially, the patient described a generalized abdominal pain, but it later had a shift to the right lower abdomen. He also mentioned nausea, vomiting, and loss of appetite. The patient had no defecation in the past 2 days. He mentioned no surgical or medical history. In addition, they did not mention any relevant medical history from his childhood.

### 2.2 Assessment

On physical examination, there was a distended abdomen, abdominal tenderness mainly localized in the right lower abdomen, and a positive rebound tenderness. Rovsing's sign was negative. In rectal exam, the rectum was collapsed and empty. The patient's vital signs were within the normal range.

**Fig. 1:**
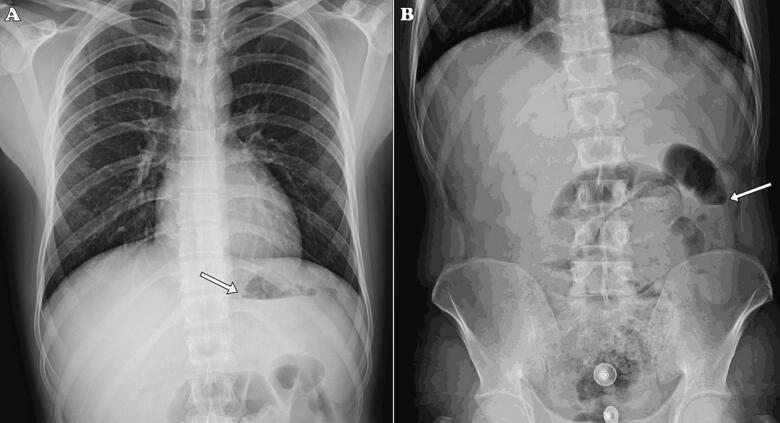
Patient's chest radiography (A) and abdominal radiography (B) with arrows indicating air-fluid levels, suggesting a potential bowel obstruction.

**Fig. 2:**
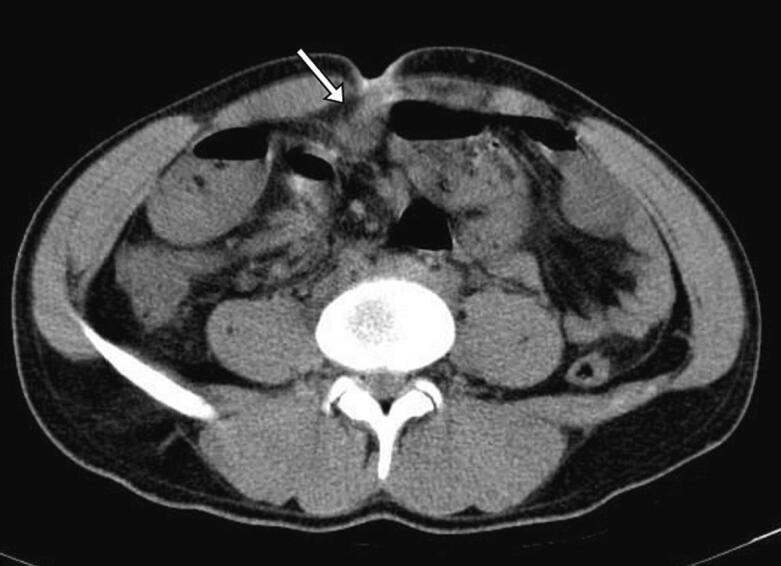
Abdominopelvic CT scan, Axial View, an arrow points to an adhesion band connecting the umbilicus to the intestinal wall, later identified as the fibrous band of the OMD.

Initially, there was suspicion of appendicitis, but obstruction was also considered. Lab test revealed a leukocytosis (WBC = 12,400) with a 90 % Neutrophil count. Abdominal ultrasonography showed mild interloop fluid, while abdominal radiography demonstrated an air-fluid level suggestive of obstruction (Fig. 1). Subsequently, a spiral abdominopelvic computed tomography scan (CT scan) was performed, revealing dilated ileal loops with a transitional zone located before the ileocecal junction. An adhesion band at this location was also observed (Fig. 2). As a result, the patient underwent laparotomy surgery because of the obstruction.

### 2.3 Operation summary

A midline lower abdominal incision was made under general anesthesia. During the explore, dissented bowel loop and an adhesion band was discovered between the umbilicus and the ileum, in about 60 cm before the ileocecal junction. The ileum and mesentery was strangulated by the fibrotic band causing the obstruction. The adhesion band resembled a fibrous band of OMD. The band was dissected and sent to the Pathology department.

### 2.4 Outcome

Following a recovery without complications, the patient was discharged. Throughout the subsequent 6-month follow-up period, the patient remained asymptomatic and did not report any complaints. Pathological investigation confirmed the presence of fibrotic tissue attached to the intestinal lumen, confirming the diagnosis of an omphalomesenteric duct remnant's fibrous band.

## 3 Discussion

The OMD remnant is a rare condition characterized by the partial or complete persistence of the embryonic OMD after birth. It is observed in approximately 2 % of infants [1]. To date, no genetic cause has been identified related to this condition [6]. Studies have shown a similar occurrence rate of OMD remnants in both males and females. However, symptomatic OMD remnants are more frequently observed in males [6,7]. Typically, different types of OMD remnants are asymptomatic. Symptoms commonly manifest when complications arise, and their presentation is age-dependent. Approximately 40 % of OMD remnants will exhibit symptoms before the age of 4, while they are rare in adults [7].

Based on the portion that remains from the embryonic OMD, there are different types of OMD remnants. Meckel's Diverticulum (MD) is characterized by a remnant portion connected to the ileum, with no umbilical connection. It is the most common form, accounting for 90 % of cases. MD is considered a true diverticulum as it contains all layers of the intestinal wall. The average age of MD presentation is 2.5 years and symptoms are uncommon in adults [1,4].

MD is mostly discovered incidentally during abdominal surgery performed for other reasons. However, MD can cause complications such as hemorrhage, bowel obstruction, inflammation (diverticulitis), and perforation [1]. Obstruction is more common in neonates and adults, while hemorrhage is the most common presentation in young children [8].

Diagnosing uncomplicated MD using imaging techniques can be challenging. Abdominal sonography and CT scan can both be utilized for diagnosis, while abdominal radiography is not particularly useful in diagnosing uncomplicated MD. While surgical intervention is unquestionably necessary in complicated MD, the management of uncomplicated MD remains controversial. Many studies argue that surgery is not essential in asymptotic cases. However, some studies suggest resecting MD when it is incidentally discovered during surgery [1,4].

The complete remnant of the OMD is known as an OMD fistula. This fistula connects the terminal ileum to the skin, and represents the second most common form of OMD remnant, accounting for approximately 10 % of symptomatic cases [9]. OMD fistulas present with the discharge of mucosal, bloody, or fecal material, leading to infection and omphalitis in infants and children. These types of OMD remnants are often responsible for cases presenting with skin abnormalities, and patients may be misdiagnosed with conditions such as pyogenic granuloma or urachal remnant. Therefore, dermatologists need to consider OMD remnant as a possibility in patients presenting with umbilical manifestations [9]. Ultrasonography can facilitate the diagnosis, as OMD fistulas can be visualized as hypoechoic tubular structures between the umbilicus and the intestinal walls. It's noteworthy that surgical intervention is necessary for the treatment of OMD fistula [1].

An OMD polyp occurs when the remaining part of the OMD is connected to the skin but closed on the other side [9]. The polyp contains mucosa and can lead to inflammation and infection. It can present with omphalitis, dermal manifestations, and similar symptoms as an OMD fistula. Ultrasonography is the preferred diagnostic method, and it is important to check for the presence of other types of OMD remnants during the ultrasonography. Surgical resection is necessary for OMD polyps, and surgeons should consider searching for any other possible OMD remnant tissues during surgery [1].

Partial closure of the OMD from both ends can result in the formation of an OMD cyst, which is not connected to the ileum or the skin [9]. These cysts are rare and seldom become symptomatic. However, in cases of infection, an OMD cyst can become symptomatic and present with firm, erythematous swelling of the skin or umbilical discharge. OMD cysts can be diagnosed using both ultrasonography and CT scan. Asymptomatic cases do not require surgical intervention, but surgery is necessary when the cyst is associated with an infection [1].

When the partial obliteration of the OMD, is resulting in a connection between the ileum and the umbilicus through a fibrous tissue, it is referred to as a fibrous band [9]. Similar to other OMD remnants, fibrous bands are commonly asymptomatic. The most significant complication associated with OMD fibrous bands is bowel obstruction, as mentioned in our case [1,3]. While abdominal ultrasonography, radiography, and abdominal CT scans can be useful in diagnosing bowel obstruction, fibrous bands can only be visualized through CT scans, and even then, detection can be challenging. Thicker fibrous bands are more likely to be detected on CT scans [1]. Therefore, there have been few reported cases in which OMD fibrous bands were diagnosed preoperatively, and the diagnosis is usually made during surgery [3].

Bowel obstruction is a common condition that requires prompt investigation. Symptoms such as abdominal pain, nausea, vomiting (particularly biliary vomiting), the absence of gas passing, and defecation difficulties can all indicate the presence of an obstruction [1,7]. Numerous conditions can cause bowel obstruction, and OMD remnant is considered a rare cause. Obstruction due to an OMD remnant is more commonly associated with Meckel diverticulum, but fibrous bands can also be responsible in certain cases. There are several mechanisms through which OMD remnants can lead to obstruction, including invagination of the MD causing intussusception, twisting of the base of the diverticulum leading to volvulus, mechanical pressure on bowel loops caused by a fibrous band or mesodiverticular band, luminal obstruction of the diverticulum due to stone or phytobezoar formation, and secondary adhesions resulting from Meckel diverticulitis [10].

Abdominal radiography is the primary diagnostic tool for obstruction, although it is unable to visualize fibrous bands. The presence of air-fluid levels is the main evidence of existing obstruction on radiography, as demonstrated in our case. Ultrasonography can be performed in cases suspected of bowel obstruction, particularly in pediatric and infant patients [1]. The presence of a pattern of fluid-filled dilated loops on ultrasonography can suggest obstruction, although the presence of intestinal gas can make high-quality imaging challenging [3]. Abdominal CT scan can also help diagnose bowel obstruction and specify the underlying etiologies. While the management of certain cases of bowel obstruction can be non-surgical, in cases where an OMD remnant is identified as the etiology of the obstruction, surgical intervention is essential [1,3,7].

## 4 Conclusion

This case highlights the importance of diagnosing and managing bowel obstruction, emphasizing the significance of identifying the underlying mechanism of obstruction for appropriate management. OMD remnant is an exceedingly rare condition in adults that can lead to bowel obstruction in certain cases. Physicians and surgeons should be aware of this rare pathology and consider it in the investigation of bowel obstruction.

## Ethical approval

At our institution, we have a specific policy regarding ethical approval for deidentified case reports. In such cases, where patient identification and sensitive information have been removed, the requirement for ethical approval is waived. This policy is overseen by the Research Ethics Committee of School of Medicine - Shahid Beheshti University of Medical Sciences.

## Funding

This article did not receive funds.

## Author contribution

Conceptualization and Methodology: Dr. Hamed Tahmasbi, Dr. Mohammad Aghaei.

Writing first draft: Dr. Arman Hasanzade, Dr. Hamed Tahmasbi, Dr. Shaghayegh Sadeghmousavi.

Investigation: Dr. Shaghayegh Sadeghmousavi.

Data curation: Dr. Alireza Haghbin Toutounchi, Dr. Mohammad Aghaei.

Writing–review & editing: all authors.

All authors read and approved the final manuscript.

## Guarantor

Dr. Mohammad Aghaei accepts all responsibility of this article.

## Declaration of competing interest

All authors declare that they have no conflicts of interest.
